# Association between Folic Acid Supplementation and Hypertensive Disorder Complicating Pregnancy in Jiangsu Province: A Cross-Sectional Study

**DOI:** 10.1155/2022/7255331

**Published:** 2022-09-06

**Authors:** Jing Cong, Danhua Pu, Rongrong Tan, Xiaoyun Ge, Weipei Zhu, Cai-e Shen, Jianfen Ge, Xiucui Luo, Juan Liu, Jie Wu

**Affiliations:** ^1^The State Key Laboratory of Reproductive Medicine, Department of Obstetrics and Gynecology, The First Affiliated Hospital of Nanjing Medical University, Nanjing Medical University, Nanjing 210029, China; ^2^Nantong Maternal and Child Health Care Hospital, Nantong 226006, China; ^3^The Second Affiliated Hospital of Soochow University, Suzhou 215008, China; ^4^Suzhou Municipal Hospital, Suzhou 215008, China; ^5^Yancheng Maternity and Child Health Care Hospital, Yancheng 224001, China; ^6^Lianyungang Maternal and Child Heath Hospital, Lianyungang 222000, China; ^7^Yangzhou Maternal and Child Care Service Centre, Yangzhou 225000, China

## Abstract

**Objectives:**

To investigate the association of folic acid (FA) supplementation with hypertensive disorder complicating pregnancy (HDCP) and preeclampsia in Jiangsu Province, China.

**Materials and Methods:**

In this cross-sectional study, a total of 10,662 women with infants born between January 2017 and December 2018 were enrolled in Jiangsu Province, China. Maternal women with and without FA supplement intake were compared in this study. FA supplementation included 0.4 mg FA (0.4 FA), multivitamins with 0.4 mg FA (multivitamin (MV)+0.4 FA), and multivitamins with 0.8 mg FA (MV + 0.8 FA). Associations between FA intake, FA supplement dose or duration, (MV + FA) dosage per weight, and HDCP were analysed using ANOVA, the chi-square test, and logistic regression analysis.

**Results:**

Over the study follow-up period, the incidences of HDCP and preeclampsia were 3.5%, 1.4%, and 2.2%, 0.6% in the non-FA supplementation and FA supplementation groups, but only 1.5% and 0.1% in the MV + 0.8 FA group in early pregnancy. Compared with the non-FA group, HDCP and preeclampsia had the lowest risk in the MV + 0.8 FA group among the seven FA supplementation groups (HDCP: RR = 0.42, 95% CI = 0.27-0.68, *P*=0.001; preeclampsia: RR = 0.09, 95% CI = 0.03–0.33, *P*=0.001) in early pregnancy. Compared with the 0.4 FA alone group, the risk of HDCP and preeclampsia in women taking MV + 0.8 FA was significantly reduced (RR = 0.60, 95% CI = 0.41–0.87, *P*=0.008; preeclampsia: RR = 0.18, 95% CI = 0.06–0.60, *P*=0.005) in early pregnancy. (MV + FA)/BMI supplementation was associated with the risk of HDCP in early pregnancy (*P* trend = 0.002).

**Conclusions:**

MV supplement with 0.8 mg FA during early pregnancy may be effective in reducing HDCP and preeclampsia risk. The study provided the viewpoint that (MV + FA)/BMI could be used as a reference for FA intake in pregnant women of different weights.

## 1. Introduction

Hypertensive disorder complicating pregnancy (HDCP), including gestational hypertension, preeclampsia, chronic hypertension complicated by preeclampsia, and chronic hypertension during pregnancy, is a group of diseases that coexist with pregnancy and hypertension [1]. The HDCP of the study was defined as new onset of hypertension arising after 20 weeks gestation, as recommended by the 2017 American College of Cardiology/American Heart Association guidelines. HDCP is the most common complication of pregnancy and significantly affects maternal and infant health. Prevalence of 3.6-9.1% and 1.4-4% has been reported for hypertension in pregnancy and preeclampsia, respectively [2]. The pathogenesis may be due to systemic vasospasm and vascular endothelial damage, followed by systemic organ perfusion reduction, which is harmful to mothers and infants [3]. Women with poor blood pressure control in early pregnancy have a significantly increased risk of target organ damage in the maternal foetus, low birth weight, preeclampsia, and other adverse outcomes [4, 5]. An epidemiological investigation revealed that a maternal age less than 20 or greater than 35 years [6], a history of preeclampsia, antiphospholipid syndrome [7], hypertension, chronic nephritis, first pregnancy, an interval of pregnancy ≥10 years, systolic blood pressure ≥130 mmHg, or diastolic blood pressure ≥80 mmHg in early pregnancy were all closely related to the occurrence of the disease. Most current recommendations for treatment are based on expert opinion and observational studies, with little evidence from randomized controlled trials. There is no effective strategy for the prevention of the disease.

Folic acid (FA), which is a water-soluble B vitamin, is widely known to prevent birth defects [8, 9]. FA is an essential cofactor involved in nucleic acid synthesis, DNA methylation and repair, cell division, and embryogenesis, making it essential for human growth and development [10, 11]. To reduce the risk of neural tube defects (NTDs) (such as spina bifida and anencephaly), the US Preventative Services Task Force (USPSTF) recommends daily FA supplementation of 0.4-0.8 mg for all women who are planning or are capable of pregnancy [12]. Studies have shown that prenatal FA supplementation significantly improves plasma and erythrocyte FA levels in women of childbearing age and reduces the risk of subsequent adverse pregnancy outcomes, such as small for gestational age (SGA) and preterm delivery [13]. Currently, there is no uniform regulation of FA supplementation dosage and duration to prevent diseases related to pregnancy. According to some studies [14, 15], FA supplements can reduce the risk of gestational hypertension and preeclampsia. The associated mechanism may be that FA supplementation reduces the homocysteine (Hcy) concentration in the blood, leading to quantitative changes in lipid and lipoprotein parameters, thereby reducing the risk of arteriosclerosis and HDCP. However, the availability of FA or folic acid-containing MV supplementation and the dose were found to be controversial in a recent meta-analysis. Therefore, this study explored the relationship between the FA supplementation dosage and time during pregnancy and HDCP and preeclampsia.

## 2. Materials and Methods

### 2.1. Study Design and Sample

Data were collected from a large hospital-based survey conducted between January 2017 and December 2018 in Jiangsu, China. In order to better evaluate the incidence of HDCP and preeclampsia in pregnant women in Jiangsu, a simple random sampling method was employed to obtain the sample, and six hospitals were randomly selected, including the Second Affiliated Hospital of Soochow University, Suzhou Municipal Hospital, Nantong Maternal and Child Health Care Hospital, Yangzhou Maternal and Child Care Service Centre, Yancheng Maternity and Child Health Care Hospital, and Lianyungang Maternal and Child Health Hospital. The participants who gave birth at a gestational age of ≥28 weeks and agreed to enrol in the project were asked to register and sign an informed consent form by two experienced doctors, and data regarding perinatal children (including live births, stillbirths, and deaths within the last 7 days) who were born were recorded from 28 weeks of gestation to 7 days postpartum. The authors declare that all the experiment protocol for involving humans was in accordance with the 2018 Declaration of Helsinki.

### 2.2. Measurements and Data Collection

**Figure 1:**
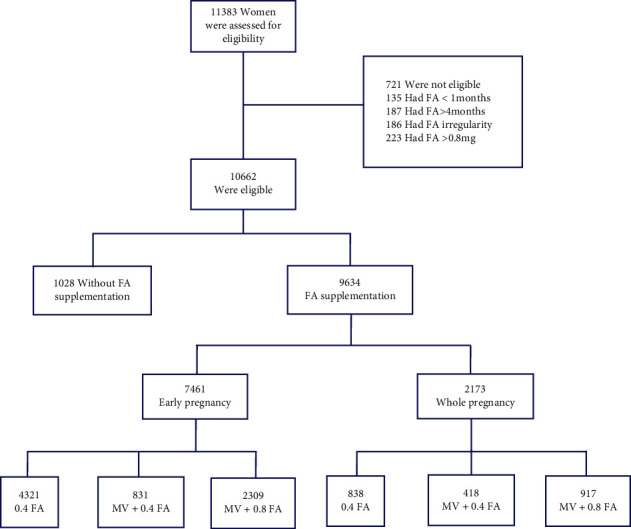
Flow diagram of recruitment and follow-up in this cohort study.

HDCP was measured at enrolment and at each of the follow-up visits by trained medical workers. Information was collected on sociodemographic characteristics (including age, ethnicity, education, marital status, address of residence, and occupation), FA supplementation information, pregnancy complications (HDCP, GDM, PTD, miscarriage, and stillbirth), health-related variables (body mass index (BMI), family history, chronic diseases, drug use, and personal history of fracture), reproductive factors (parity and menstrual status), and maternal exposure to perinatal risk factors (including lifestyle, illnesses, perinatal stress, exposure to harmful factors in the perinatal environment, and family history of diseases). The periconceptional weight was recorded on a perinatal care card in the first trimester by a doctor at a perinatal care centre. Each participant underwent a face-to-face interview in which trained physicians at a maternity or postpartum ward administered field surveys using a structured questionnaire. Details regarding FA supplementation, including the formulation, daily dosage, and starting and ending times of FA supplementation, were collected at enrolment. 11,383 pregnant women were screened, and 721 women were excluded due to irregularities or mismatched conditions in FA supplementation. Of the remaining 10,662 pregnancies, 1,028 of the participants did not take any FA supplement and 9634 took FA supplement. According to the duration of FA supplementation, 7,461 pregnant women supplemented in early pregnancy, and 2,173 women supplemented throughout pregnancy. Based on the dosage of FA supplementation, 9,634 of the enrolled women were divided into groups receiving 0.4 mg/d FA alone (*n* = 4,321), multivitamins (MVs) with 0.4 mg/d FA (*n* = 831), or MVs with 0.8 mg/d FA (*n* = 2,309) in early pregnancy and 0.4 mg/d FA alone (*n* = 838), MVs with 0.4 mg/d FA (*n* = 418), or MVs with 0.8 mg/d FA (*n* = 917) in whole pregnancy, as shown in Figure 1. This study was approved on 10/1/2017 by the institutional Ethics Committee of the First Affiliated Hospital of Nanjing Medical University (2016-SR-231).

### 2.3. Statistical Analysis

Quantitative variables are expressed as the means ± standard deviations (SDs) or medians, and qualitative variables are expressed as percentages. The chi-square test and ANOVA were used to compare categorical and continuous data, respectively. Logistic regression analysis was employed to assess the unadjusted and adjusted relative risks (RR) and their 95% CI between the timing of folic acid-containing supplement use and the outcomes. There are considerable differences in the weights of pregnant women, which are associated with individual metabolic rates. In this cross-sectional study, the logistic regression model showed that the incidence of pregnancy complications was associated with weight. To avoid the influence of differing prepregnancy weights between individuals, the study introduced the (MV + FA) dosage per BMI to evaluate the relationship between FA supplementation and HDCP. In this study, (MV + FA)/BMI was categorized into 4 groups according to quartiles. A linear trend test was conducted by assigning ordinal values 1, 2, 3, and 4 to *Q*1, *Q*2, *Q*3, and *Q*4, respectively. Binary logistic regression and adjusted relative risks (RRs) in the population were used. The statistical software package SPSS Statistics 23.0 (SPSS Inc., Chicago, IL, USA) was used for data analyses.

## 3. Results

### 3.1. Participants

The flow diagram of recruitment and follow-up in this cohort study is shown in Figure 1. A comparison of the baseline characteristics of the participants based on FA supplementation status is shown in Table 1, including sociodemographic characteristics, pregnancy complications, HDCP, GDM, age, prepregnancy BMI, and maternal education.

### 3.2. Relationship between FA Supplementation and HDCP and Preeclampsia

**Figure 2:**
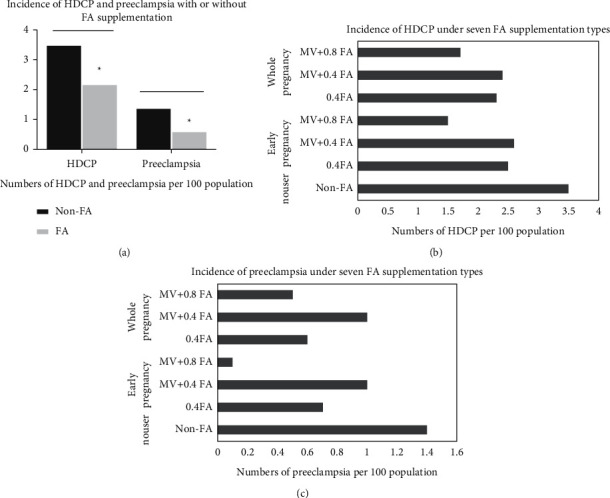
Incidence of HDCP and preeclampsia in Jiangsu Province. (a) Incidence of HDCP and preeclampsia with and without FA supplementation. (b) Incidence of HDCP under seven FA supplementation types. (c) Incidence of preeclampsia under seven FA supplementation types. FA: folic acid, Nonuser: non-FA, 0.4 FA: 0.4 mg folic acid, MV + 0.4 FA: multivitamins with 0.4 mg folic acid, MV + 0.8 FA: multivitamins with 0.8 mg folic acid. HDCP: hypertensive disorder complicating pregnancy, GDM: gestational diabetes mellitus. ^*∗*^*P* < 0.05.

The incidence of HDCP and preeclampsia in the FA supplementation group was significantly lower than that of the non-FA supplementation group (Figure 2(a)). For clarity, the study used a histogram to describe the incidence of HDCP and preeclampsia after seven FA supplementation modes (Figures 2(b) and 2(c)). As shown in Table 2, the incidences of HDCP and preeclampsia were 3.5%, 1.4%, and 2.2%, 0.6% in the non-FA supplementation and FA supplementation groups, but only 1.5% and 0.1% in the MV + 0.8 FA group in early pregnancy. In the logistic regression model, compared with the non-FA group, Table 3 shows that the lowest risk of HDCP and preeclampsia occurred in the MV + 0.8 FA group in early pregnancy among the seven FA supplementation groups (HDCP: RR = 0.42, 95% CI = 0.27-0.68, *P*=0.001; preeclampsia: RR = 0.09, 95% CI = 0.03-0.33, *P*=0.001) without adjusting for any potential covariates. The risk of HDCP and preeclampsia in the MV + 0.8 FA group in early pregnancy was still lowest after adjusting for education, prepregnancy BMI, age, weight gain during pregnancy, ethnicity, infant gender, and primiparity (HDCP: RR = 0.49, 95% CI = 0.31-0.80, *P*=0.004; preeclampsia: RR = 0.11, 95% CI = 0.03-0.39, *P*=0.001). When compared with the 0.4 FA group in the early pregnancy and whole pregnancy, respectively, Table 4 shows that the lowest risk of HDCP and preeclampsia occurred in the MV + 0.8FA group among the three FA supplementation groups (HDCP: RR = 0.60, 95% CI = 0.41-0.87, *P*=0.008; preeclampsia: RR = 0.18, 95% CI = 0.06-0.60, *P*=0.005) without adjusting for any potential covariates and (HDCP: RR = 0.60, 95% CI = 0.41-0.88, *P*=0.009; preeclampsia: RR = 0.18, 95% CI = 0.06–0.59, *P*=0.005) after adjusting for education, prepregnancy BMI, age, weight gain during pregnancy, ethnicity, infant gender, and primiparity in early pregnancy.

### 3.3. Relationship between (MV + FA)/BMI Supplementation and HDCP

(MV + FA)/BMI supplementation was inversely associated with HDCP after adjusting for potential covariates, such as education, prepregnancy BMI, age, weight gain during pregnancy, ethnicity, infant gender, and primiparity. As shown in Table 5, in early pregnancy, the RRs of HDCP across increasing quartiles of (MV + FA)/BMI supplementation intake were 1.00 (reference) (RR = 0.60, 95% CI = 0.31-1.15) for *Q*2, (RR = 0.52, 95% CI = 0.26-1.07) for *Q*3, and (RR = 0.28, 95% CI = 0.12-0.65) for Q4 (*P* trend = 0.002). However, throughout pregnancy, (MV + FA)/BMI supplementation was not associated with the risk of HDCP (*P* trend = 0.13).

## 4. Discussion

Prenatal FA supplementation can reduce congenital neural tube defects is a well-known fact. Because of this important role of FA, many countries have introduced corresponding policies to encourage women who are planning to become pregnant or are already pregnant to take FA. Since 2009, the “Folic Acid Supplement Project” has been implemented in China and resulted in a high supplementation rate (9634/10662) among the general public. As a public health project, FA supplementation during pregnancy has attracted public attention, and there are many different FA supplements in the market with different formulations and dosages, including MVs containing FA and FA-only supplements. With the in-depth research on FA supplementation, the relationship between FA supplementation during peripregnancy and some pregnancy-related diseases has also received public attention, especially HDCP. The study analysed the most common types of FA supplements in the market that are taken by women who are planning to become pregnant or are already pregnant, with the aim of identifying the optimal supplement combination to prevent HDCP and preeclampsia.

This study showed that FA supplementation can significantly reduce the incidence of HDCP and preeclampsia in Jiangsu Province, especially in the MV + 0.8 FA group in early pregnancy, compared with the non-FA group. This result was supported by Wang's et al. study [16], which provided evidence that FA supplementation and higher dietary folate intake during pregnancy reduce the risk of preeclampsia. The benefits of FA supplementation in early pregnancy have also been supported by several studies [17, 18]. Martinussen's et al. study [19] supports a possible protective effect of folate intake in early pregnancy against preeclampsia in lean mothers. Meanwhile, some studies [20, 21] also found that supplementation with MVs containing FA was beneficial for the reduction of HDCP. However, this study showed that MVs containing 0.8 mg FA reduced HDCP risk, while MVs containing 0.4 mg FA did not. A study [22] similar to this conclusion showed that intake of 0.4 mg FA alone per day during early pregnancy cannot prevent the occurrence of gestational hypertension and preeclampsia. At present, there are some research studies on the mechanism of FA in preventing HDCP. A randomized clinical trial [23] provided evidence that a high dosage of FA supplements throughout pregnancy reduces Hcy concentrations at the time of delivery and further reduces preeclampsia. High levels of Hcy in the blood may lead to the occurrence of gestational hypertension. Studies [23] have shown that FA may reduce the risk of hypertension by lowering the Hcy level in the blood. FA can decrease the DNA damage and cell apoptosis caused by oxidative free radicals and some metabolites by reducing the Hcy concentration in serum. FA supplementation may affect placental implantation and vascular remodeling by influencing the process of DNA and protein synthesis and antioxidant defences. FA is involved in many biological processes in the body and plays an important role in the process of biological and nervous system development and function [24]. Of course, the research on the mechanism of FA in reducing HDCP and preeclampsia still needs further verification in vivo and in vitro.

However, other studies have shown that FA supplementation throughout pregnancy also yields other benefits [15, 25]. Other studies [26] have shown that FA supplementation and higher dietary folate intake during preconception and pregnancy decrease the risk of preterm birth, with the protective effect varying by preterm subtype. Some Western countries have even fortified their diets with FA to increase FA intake. However, there are some other viewpoints in the research on FA supplementation. Excessive daily FA intake during pregnancy was seen among mothers of positional plagiocephaly patients. Women who did not take FA and who took FA for >90 days had a higher incidence of GDM than those who took FA for ≤60 days [27]. Long-term, high-dose, daily supplementation with FA, and vitamins B6 and B12 did not reduce the overall depression risk in mid-life and older women [28]. Therefore, FA supplementation benefits are time-limited. In this study, MV + 0.8 FA supplementation per day during early pregnancy reduced the HDCP risk. A meta-analysis in South Korea showed that taking FA alone or an MV with FA did not significantly reduce the risk of gestational hypertension or preeclampsia [29]. It may be that the FA supplementation dosage and duration in the six studies included in this meta-analysis were not completely consistent, and the heterogeneity was large. Since the importance of FA supplementation is obvious, disputes regarding the dosage and time may be the result of many factors, such as different regions, diets, and environments. Regarding this conclusion, MV + 0.8 FA supplementation per day during early pregnancy reduced the HDCP and preeclampsia risk that requires a better-designed, large-sample, randomized controlled trials to verify. However, more literature supports the notion that FA supplementation during pregnancy can reduce preeclampsia.

There are considerable differences in the BMI among pregnant women, which are associated with differences in individual metabolic rates. To avoid the influence of the prepregnancy BMI on the FA supplement dosage, reduce individual differences, and improve the accuracy of the results, the logistic regression model used in this cross-sectional study showed that the incidence of pregnancy complications was associated with BMI. Considering the above reasons, the (MV + FA) dosage per BMI was used to evaluate the relationship between FA supplementation and HDCP. Interestingly, good correlations were found between the results of (MV + FA)/BMI supplementation and HDCP. Although the sensitivity and specificity were not very high and a large number of clinical studies are needed for verification, this study provides a basis for exploring individual FA supplementation doses in the future. However, the study has limitations. First, the FA exposure was measured according to selfreported FA supplement use rather than plasma folate measurement. In order to maintain the authenticity of the data, we checked by referring to the perinatal care card of pregnant women. For those in doubt, we also needed to consult the family of pregnant women for confirmation. A small sample survey was conducted early in this study and found that selfreported folic acid supplementation was associated with plasma folic acid and was considered a reliable measure of folic acid exposure. Second, the study discussed the relationship between FA supplementation and pregnancy-induced hypertension, which was not further classification of the different types of hypertension.

## 5. Conclusion

MV supplement with 0.8 mg FA during early pregnancy may be effective in reducing HDCP and preeclampsia risk. The study provided the viewpoint that (MV + FA)/BMI could be used as a reference for FA intake in pregnant women of different weights.

## Figures and Tables

**Table 1:** General participant characteristics.

	Nonuser	User						
	Non-FA (*n* = 1028)	Total (*n* = 9634)	Early pregnancy			Whole pregnancy		
			0.4 FA (*n* = 4321)	MV + 0.4 FA (*n* = 831)	MV + 0.8 FA (*n* = 2309)	0.4 FA (*n* = 838)	MV + 0.4 FA (*n* = 418)	MV + 0.8 FA (*n* = 917)
Age at pregnancy, yrs (mean ± SD)	30.63 ± 4.97	30.08 ± 3.98	30.10 ± 4.05	29.89 ± 3.83	29.56 ± 3.88	30.41 ± 3.99	30.27 ± 3.81	31.09 ± 3.86
Body mass index, kg/m2 (mean ± SD)	21.88 ± 3.23	21.44 ± 3.01	21.51 ± 3.04	21.34 ± 2.99	21.40 ± 3.04	21.42 ± 2.93	21.49 ± 3.08	21.32 ± 2.88
Han ethnicity *N* (%)	1014 (98.64)	9525 (98.87)	4264 (98.68)	819 (98.56)	2284 (98.92)	832 (99.28)	414 (99.04)	912 (99.45)
Education level ≥12 yrs *N* (%)	587 (57.10)	7326 (76.04)	3139 (72.65)	682 (82.07)	1708 (73.97)	658 (78.52)	358 (85.65)	781 (85.17)
Primiparous *N* (%)	522 (50.78)	6592 (68.42)	2817 (65.19)	606 (72.92)	1644 (71.20)	539 (64.32)	320 (76.56)	666 (72.63)
Infant gender (male/female) *N* (%)	532 (51.75)	5016 (52.07)	2254 (52.16)	432 (51.99)	1130 (48.94)	419 (50.00)	237 (56.70)	544 (59.32)
Pregnancy complications *N* (%)	201 (19.55)	1612 (16.73)	778 (18.01)	124 (14.92)	281 (12.17)	187 (22.32)	65 (15.55)	177 (19.30)
HDCP *N* (%)	36 (3.50)	211 (2.19)	109 (2.52)	22 (2.65)	35 (1.52)	19 (2.27)	10 (2.39)	16 (1.74)
Preeclampsia *N* (%)	14 (1.36)	56 (0.58)	31 (0.72)	8 (0.96)	3 (0.13)	5 (0.60)	4 (0.96)	5 (0.55)
GDM *N* (%)	100 (9.73)	1014 (10.53)	457 (10.58)	72 (8.66)	205 (8.88)	120 (14.32)	43 (10.29)	117 (12.76)
Cigarette smoking *N* (%)	147 (14.30)	1453 (15.08)	665 (15.39)	121 (14.56)	286 (12.39)	165 (19.67)	61 (14.59)	155 (16.90)
Alcohol drinking *N* (%)	29 (2.82)	352 (3.65)	147 (3.40)	43 (5.17)	78 (3.38)	30 (3.58)	27 (6.46)	27 (2.94)

FA: folic acid, 0.4 FA: 0.4 mg folic acid, MV + 0.4 FA: multivitamins with 0.4 mg folic acid, MV + 0.8 FA: multivitamins with 0.8 mg folic acid. HDCP: hypertensive disorder complicating pregnancy, GDM: gestational diabetes mellitus.

**Table 2:** Incidence of HDCP and preeclampsia in Jiangsu Province.

	Nonuser	User						
	Non-FA (*n* = 1028)	Total (*n* = 9634)	Early pregnancy			Whole pregnancy		
			0.4 FA (*n* = 4321)	MV + 0.4 FA (*n* = 831)	MV + 0.8 FA (*n* = 2309)	0.4 FA (*n* = 838)	MV + 0.4 FA (*n* = 418)	MV + 0.8 FA (*n* = 917)
HDCP								
Mean	0.035	0.022	0.025	0.026	0.015	0.023	0.024	0.017
95% CI	0.024-0.046	0.019-0.025	0.021-0.030	0.016-0.037	0.010-0.020	0.013-0.033	0.009-0.039	0.009-0.026

*Preeclampsia*								
Mean	0.014	0.006	0.007	0.010	0.001	0.006	0.010	0.005
95% CI	0.007-0.021	0.004-0.007	0.005-0.010	0.003-0.016	0.000-0.003	0.001-0.011	0.000-0.019	0.001-0.01

FA: folic acid, 0.4 FA: 0.4 mg folic acid, MV + 0.4 FA: multivitamins with 0.4 mg folic acid, MV + 0.8 FA: multivitamins with 0.8 mg folic acid. HDCP: hypertensive disorder complicating pregnancy.

**Table 3:** Regression analysis of FA supplementation with HDCP and preeclampsia (nonusers group as reference).

	Nonuser	User					
	Non-FA (*n* = 1028)	Early pregnancy			Whole pregnancy		
		0.4 FA (*n* = 4321)	MV + 0.4 FA (*n* = 831)	MV + 0.8 FA (*n* = 2309)	0.4 FA (*n* = 838)	MV + 0.4 FA (*n* = 418)	MV + 0.8 FA (*n* = 917)
HDCP							
Crude RR (95% CI)	1	0.71(0.49-1.05)	0.75 (0.44-1.28)	0.42 (0.27-0.68)	0.64(0.36-1.12)	0.68 (0.33-1.37)	0.49 (0.27-0.89)
*P*		0.084	0.294	0.001	0.120	0.279	0.019
Model RR (95% CI)	1	0.82(0.55-1.20)	0.93 (0.54-1.61)	0.49 (0.31-0.80)	0.77(0.43-1.35)	0.80 (0.39-1.65)	0.62 (0.34-1.15)
*P*		0.305	0.789	0.004	0.358	0.553	0.128

*Preeclampsia*							
Crude RR (95% CI)	1	0.52(0.28-0.99)	0.70 (0.29-1.69)	0.09 (0.03-0.33)	0.44(0.16-1.21)	0.70 (0.23-2.14)	0.40 (0.14-1.11)
*P*		0.046	0.431	0.001	0.111	0.531	0.077
Model RR (95% CI)	1	0.61(0.32-1.16)	0.88 (0.36-2.16)	0.11 (0.03-0.39)	0.53(0.19-1.49)	0.84 (0.27-2.62)	0.53 (0.19-1.50)
*P*		0.132	0.787	0.001	0.227	0.766	0.23

Crude: without adjusting for any covariates. Model: adjusted for education, prepregnancy BMI, age, weight gain during pregnancy, ethnicity, infant gender, and primiparity. FA: folic acid, 0.4 FA: 0.4 mg folic acid, MV + 0.4 FA: multivitamins with 0.4 mg folic acid, MV + 0.8 FA: multivitamins with 0.8 mg folic acid. ^*∗*^*P* < 0.05.

**Table 4:** Regression analysis of FA supplementation and HDCP and preeclampsia (0.4FA group as reference).

	Early pregnancy (*n* = 7461)			Whole pregnancy (*n* = 2173)		
	0.4 FA (*n* = 5177)	MV + 0.4 FA (*n* = 1274)	MV + 0.8 FA (*n* = 3183)	0.4 FA (*n* = 5177)	MV + 0.4 FA (*n* = 1274)	MV + 0.8 FA (*n* = 3183)
HDCP						
Crude RR(95% CI)	1	1.05 (0.66-1.67)	0.60 (0.41-0.87)	1	0.95 (0.49-1.82)	0.67 (0.40-1.17)
*P*		0.834	0.008		0.871	0.163
Model RR(95% CI)	1	1.12 (0.70-1.79)	0.60 (0.41-0.88)	1	0.98 (0.50-1.90)	0.77 (0.45-1.31)
*P*		0.635	0.009		0.942	0.337

*Preeclampsia*						
Crude RR(95% CI)	1	1.35 (0.62-2.94)	0.18 (0.06-0.60)	1	1.34 (0.47-3.81)	0.76 (0.29-1.96)
*P*		0.457	0.005		0.586	0.568
Model RR(95% CI)	1	1.42 (0.65-3.12)	0.18 (0.06-0.59)	1	1.38 (0.48-3.95)	0.85 (0.33-2.20)
*P*		0.381	0.005		0.551	0.735

Crude: without adjusting for any covariates. Model: adjusted for education, prepregnancy BMI, age, weight gain during pregnancy, ethnicity, infant gender, and primiparity. FA: folic acid, 0.4 FA: 0.4 mg folic acid, MV + 0.4 FA: multivitamins with 0.4 mg folic acid, MV + 0.8 FA: multivitamins with 0.8 mg folic acid. ^*∗*^*P* < 0.05.

**Table 5:** Relationship between (MV + FA)/BMI supplementation dose and HDCP.

(MV + FA)/BMI	First trimester				Whole pregnancy			
	Case	Controls	RR	95% CI	Case	Controls	RR	95% CI
*Q*1 (4.26-8.33)	7	803	1		2	273	1	
*Q*2 (8.34-13.33)	12	730	0.6	0.31-1.15	6	316	1.09	0.42-2.87
*Q*3 (13.34-15.38)	16	848	0.52	0.26-1.07	9	375	0.79	0.28-2.25
*Q*4 (15.39-21.05)	22	702	0.28	0.12-0.65	8	305	0.31	0.06-1.42
Total	57	3083			25	1269		
*P* Trend			0.00				0.13	

^*∗*^: adjusted for education, prepregnancy BMI, age, weight gain during pregnancy, ethnicity, infant gender, and primiparity. (MV + FA)/BMI: (MVs + FA)/BMI, HDCP: hypertensive disorder complicating pregnancy; case: HDCP, controls: non-HDCP.

## Data Availability

The data used to support the findings of this study are available from the corresponding author upon request.

## Abbreviations

FA: Folic acid
HDCP: Hypertensive disorder complicating pregnancy
GDM: Gestational diabetes mellitus
BMI: Body mass index
Hcy: Homocysteine
RR: Relative risk.

## Additional Points

The authors declare that the experiment protocol for involving humans was in accordance with the 2018 Declaration of Helsinki.
